# Cross-Modal Augmented Transformer for Automated Medical Report Generation

**DOI:** 10.1109/JTEHM.2025.3536441

**Published:** 2025-01-29

**Authors:** Yuhao Tang, Ye Yuan, Fei Tao, Minghao Tang

**Affiliations:** Jiangsu Police Institute 164369 Nanjing 210031 China; Jiangsu Provincial Branch of the Industrial and Commercial Bank of China Nanjing 210006 China; Yangzhou Intermediate People’s Court of Jiangsu Province Yangzhou 225009 China; The First People’s Hospital of Jiashan Jiaxing 314100 China

**Keywords:** Medical report generation, medical imaging, automatic diagnosis, clinical automation, image captioning

## Abstract

In clinical practice, interpreting medical images and composing diagnostic reports typically involve significant manual workload. Therefore, an automated report generation framework that mimics a doctor’s diagnosis better meets the requirements of medical scenarios. Prior investigations often overlook this critical aspect, primarily relying on traditional image captioning frameworks initially designed for general-domain images and sentences. Despite achieving some advancements, these methodologies encounter two primary challenges. First, the strong noise in blurred medical images always hinders the model of capturing the lesion region. Second, during report writing, doctors typically rely on terminology for diagnosis, a crucial aspect that has been neglected in prior frameworks. In this paper, we present a novel approach called Cross-modal Augmented Transformer (CAT) for medical report generation. Unlike previous methods that rely on coarse-grained features without human intervention, our method introduces a “locate then generate” pattern, thereby improving the interpretability of the generated reports. During the locate stage, CAT captures crucial representations by pre-aligning significant patches and their corresponding medical terminologies. This pre-alignment helps reduce visual noise by discarding low-ranking content, ensuring that only relevant information is considered in the report generation process. During the generation phase, CAT utilizes a multi-modality encoder to reinforce the correlation between generated keywords, retrieved terminologies and regions. Furthermore, CAT employs a dual-stream decoder that dynamically determines whether the predicted word should be influenced by the retrieved terminology or the preceding sentence. Experimental results demonstrate the effectiveness of the proposed method on two datasets.Clinical impact: This work aims to design an automated framework for explaining medical images to evaluate the health status of individuals, thereby facilitating their broader application in clinical settings.Clinical and Translational Impact Statement: In our preclinical research, we develop an automated system for generating diagnostic reports. This system mimics manual diagnostic methods by combining fine-grained semantic alignment with dual-stream decoders.

## I. Introduction

**FIGURE 1.**
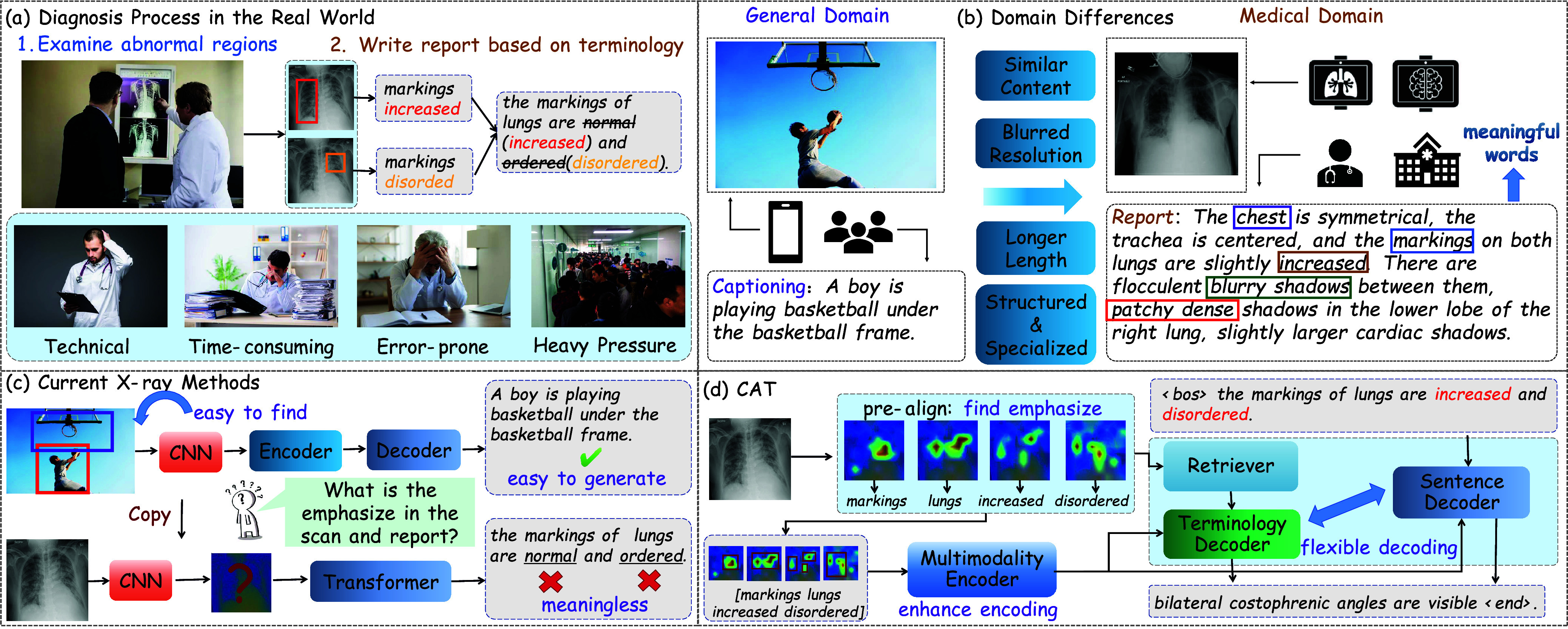
The current diagnostic process worldwide is not only time-consuming and labor-intensive but also susceptible to errors. Medical datasets often exhibit notable data biases, and existing X-ray methods may fail to capture rare yet significant abnormalities, resorting instead to generating template sentences. In contrast, the proposed model surpasses baseline methods that only produce normal descriptions by generating more comprehensive abnormal descriptions.

Radiology image is globally the most prevalent form of medical imaging and plays a crucial role in detecting prevalent thoracic conditions such as lung cancer and pneumonia [1]. It enables doctors to meticulously analyze complex images with the aim of identifying the presence and condition of abnormalities [2]. During this process, doctors are required to identify specific regions, and subsequently generate detailed reports pertaining to these regions. These reports serve as essential components of the diagnostic process, providing a thorough description of the depicted organs and their normality. They act as compelling evidence, aiding doctors in formulating final diagnostic conclusions. However, as depicted at the Fig. 1(a), it is crucial to acknowledge that identifying salient regions from blurred images and then crafting detailed reports can prove to be a labor-intensive and error-prone task. This situation is further exacerbated by the prevalent shortage of adequately trained medical professionals in healthcare systems. The scarcity of human resources poses a challenge to the efficiency and effectiveness of medical image examinations, potentially leading to delays in diagnosis and treatment. In light of these challenges, the development of a model capable of automatically diagnosing medical images and generating comprehensive reports holds tremendous potential.

To discuss medical report generation, it is important to briefly introduce image captioning, as it is closely related to medical report generation. In traditional image captioning task, an open-structured sentence is automatically generated to succinctly describe the visual content depicted in the images. A fundamental image captioning model typically adheres to a unified paradigm, which involves utilizing a Convolutional Neural Network (CNN) as an encoder to represent visual features, and employing a Recurrent Neural Network (RNN) as a decoder to generate pertinent textual descriptions [3], [4]. Extensive research efforts in this domain revolve around augmenting both the encoder and decoder components by integrating meticulously designed attention mechanisms [5], [6], [7], enhancing transformer-based architectures [8], [9], [10], and harnessing the power of additional language models [11], [12]. Due to the advancements resulting from these state-of-the-art studies, medical report generation has garnered substantial attention within the research community. Numerous existing methodologies, such as those proposed in [13], [14], [15], and [16], closely align with standard image captioning approaches and effectively leverage the prevalent encoder-decoder framework.

Despite significant advancements in image captioning, a noticeable decline in performance is frequently observed when directly applying image captioning models to medical report generation, as illustrated at the Fig. 1(b) and (c). This difference can be attributed to two primary factors:

***(i) Medical images inherently exhibit higher levels of ambiguity compared to images in the general domain, posing challenges in describing intricate regions that bear clinical significance.*** Given that not all regions depicted in a medical image hold textual relevance or convey explicit language cues, it becomes imperative to accurately pinpoint disease-specific areas within these images. Some methodologies entail the utilization of classifier models to predict specific diseases [15], [17]. However, these models typically rely on task-specific manual annotations, thereby constraining their applicability across various scenarios. Moreover, the scope of information encompassed within these annotations is often limited, resulting in relatively weak associations between image regions and medical terminologies.

***(ii) Despite the rising popularity of Transformer-based architectures in medical report generation, they are accompanied by increased textual data variance.*** In medical reports, it is common for physicians to provide comprehensive descriptions of all elements present in a medical image, often resulting in an overemphasis on detailing normal regions within the report. Moreover, the repetitive use of similar sentences to describe these normal areas further compounds the issue of textual imbalance. This skewed distribution of textual data during training can lead to the generation of an excessive number of sentences pertaining to normal findings, thereby hindering the model’s ability to effectively convey critical medical terminology. In fact, current approaches tend to produce redundant descriptions of normal findings while overlooking the inclusion of rare yet significant medical terms. Limited research efforts have been directed towards leveraging these medical terminologies in conjunction with Transformer-based decoders to devise innovative architectures for report generation.

To capture rare yet crucial abnormal regions within medical images, a primary challenge arises in the initial alignment of images and textual descriptions prior to elucidating the image contents. Furthermore, the integration of medical terminologies to facilitate more adaptable generation processes is a crucial step towards making the model more suitable for medical scenarios. As depicted in the Fig. 1(d), we introduce the Cross-modal Augmented Transformer (CAT) framework. In the typical clinical setting, medical professionals meticulously scrutinize abnormal regions depicted in medical images and subsequently attribute descriptive interpretations to these regions. Drawing upon extensive medical expertise, they proceed to craft corresponding reports. To mirror this procedural workflow, we introduce a Keyword-Region Pre-alignment (KRP) module aimed at aligning essential medical terminologies with critical regions. Rather than utilizing the entire report as the learning objective, our approach involves extracting abnormal terms as labels to underscore regions of substantive relevance and preserving those with heightened significance. Additionally, we enhance the encoder by incorporating the cross-modal embeddings of retrieved terminologies, predicted keywords and critical regions. To improve the generation performance, we leverage two separate stream decoders to decode terminologies and generated sentences as an alternative to the single stream decoder. The current word prediction is contingent upon the terminologies derived from the KRP module and the generated sentence branch. Through this mechanism, the decoder leverages the interplay between visual cues and diverse textual contexts to foster a more adaptive generation of target reports.

In summary, the contributions are listed as follows:

• To better describe fine-grained regions that are clinically significant, we introduce a Keyword-Region Pre-alignment module(KRP). This module focuses on aligning key medical words with critical regions and capturing the vital features by excluding those with weaker correlations. This operation provides features with reduced noise to the report generation framework, facilitating the generation of reports that closely resemble the ground truth.
• To enhance the relationship between medical terms and specific regions, a cross-modal retriever is designed to find more informative reports. Subsequently, retrieved reports, generated keywords, and visual features are integrated to enhance the Multimodality Encoder(ME).
• Additionally, we replace the Transformer-based decoder with separate branches for terminology and sentences. This Dual-Stream Decoder (DSD) leverages retrieved terminologies to augment the capabilities of the traditional decoder. It operates cross-modal self-attention interaction between the two branches.
• The experiments and analyses conducted on two public datasets demonstrate that the proposed method achieves competitive performance, aligning more closely with the requirements of clinical applications.

A citation example was provided in the previous sentence.

## II. Related Work

### A. Image Captioning

Image Captioning involves automatically generating a descriptive sentence for a given image. Over the past few years, numerous methods have been proposed, leading to significant advancements in the field [4], [5], [6], [18], [19], [20], [21], [22], [23]. Most of these methods utilize the conventional encoder-decoder architecture, where a Convolutional Neural Network (CNN) serves as the encoder, and a Recurrent Neural Network (RNN) acts as the decoder with an attention mechanism to generate the sentence [24]. Building upon this framework, several methods have introduced the attention mechanism [4], [18], [19]. By incorporating the attention mechanism, these models are able to automatically learn to focus on vital regions while generating the corresponding words. In addition to developing attention mechanisms, other works in image captioning also focus on advancing the encoder and decoder. Some approaches explicitly consider structural semantics among visual objects by constructing scene graphs [20], [21] or graphical convolutional networks [22]. Some studies also aim to improve the text decoder. The Transformer model has been proposed to augment the limited representation capacity of RNNs and has been applied in image captioning tasks to replace RNNs as the text decoder [8], [25].

Despite achieving promising performance, the uninformed application of these image captioning methods from general domains negatively impacts the performance of medical report generation. It is crucial to take into account the significant distinctions between the situations in the general domain and medical domain. Firstly, medical reports are typically more complex and longer compared to descriptions in the general domain, as they describe multiple pathological areas within an image. Instead of producing a single sentence, the goal of medical report generation is to generate a lengthy paragraph comprising multiple structured sentences, each dedicated to a specific medical observation for a particular region within the image. Secondly, medical images exhibit more fine-grained characteristics, making it challenging to locate vital abnormalities. This challenge is exacerbated by the heavy data bias towards normal regions in medical imaging datasets. In a medical report, the accuracy of depicting abnormalities should be prioritized over normal findings, unlike in a general image description where each sentence holds equal weight.

### B. Medical Report Generation

Drafting a medical report can prove to be a laborious and meticulous task for seasoned doctors, and error-prone for those lacking experience. Similar to image captioning, many current studies strive to utilize conventional encoder-decoder structure to autonomously craft a coherent report [15], [26], [27], [28], [29], [30], [31]. Nonetheless, owing to significant data discrepancies, these models struggle in pinpointing visual references and tend to exhibit a bias towards generating plausible yet generic reports, often lacking in distinctive abnormal narratives. Hence, Jing et al. [32] enhance sentence generation by employing two distinct RNNs—termed Normality Writer and Abnormality Writer—instead of a single RNN for both normal and abnormal sentence generation. Yang et al. [33] introduce a hierarchical retrieval mechanism designed to autonomously extract templates at both the report and sentence levels, leveraging these extracted texts to generate comprehensive reports. However, the effectiveness of data-driven RNNs in modeling abnormal sentences is often compromised by the rarity and diversity of abnormalities.

Thanks to the remarkable achievements of Transformer in the vision-and-language domain, several Transformer-based architectures [13], [17], [34], [35] have emerged as potential candidates to supplant the RNN architecture. Chen et al. [13] introduce a relational memory mechanism designed to retain information regarding the previous generation procedure. They integrate this relational memory directly into the Transformer architecture to facilitate and enhance the decoding process. However, Liu et al. [34] believe that incorporating retrieved reports and medical knowledge graph is crucial to mimic prior working experience. By employing distinct Transformer branches, they effectively explore and distill both posterior and prior knowledge to enhance the generation of radiology reports. Conversely, Tanida et al. [17] emphasize feature encoding by concentrating on abnormal regions. They extract specific region visual features through object detection and enhance these features with an abnormality classification module for richer abnormal information. Subsequently, a pre-trained Transformer-based language model is employed as the decoder. Fan et al. [36] design a disease indicator to evaluate the states of each disease present in the scans, subsequently embedding these disease states for further generation. However, this approach necessitates manual annotation of all abnormal information within the reports, and the discrete classification fails to consider the logical relationships within the context. Specifically, different diseases need to be organized into coherent sentences. Additionally, a significant amount of noise in medical images may hinder the effectiveness of classification. Fan et al. [37] have rethought medical report generation as a multi-label classification task, where they present a generation framework that integrates BLIP with classified key nodes. This method shifts the focus of report generation beyond simply generating coherent text from scans to identifying and classifying relevant medical concepts that can be directly utilized to produce reports. This method also requires extensive category labeling of the dataset. Furthermore, we argue that classifying medical concepts is more challenging than generating them. This is because classification approaches overlook the contextual relationships between concepts, making it difficult to accurately predict a wide range of terms. Jin et al. [38] propose a cross-modal feature enhancement approach that utilizes a retrieval network. The retrieval network leverages multi-modal knowledge from a pre-trained foundation model to retrieve similar records, and the retrieved reports are then used to assist in disease classification. However, directly using blurry images to retrieve reports can introduce significant visual noise. Therefore, it is essential to consider how to perform fine-grained denoising on medical image features before feeding them into the retrieval module. Wang et al. [39] argue that in clinical scenarios, multiple medical images with different views are often generated simultaneously and have high semantic consistency. Based on this idea, they propose a multi-view contrastive learning strategy to help a deep reinforcement learning-based model leverage the consistency of multi-view inputs for better report generation learning. However, most medical datasets typically consist of a single image corresponding to a single report, rather than multiple images with different views. Furthermore, it is unfair to compare a model trained on medical images of multi-view patients with one trained on single-perspective images. Xiao et al. [40] optimize the generation framework using multi-objective reinforcement learning, achieving promising performance; however, this approach also increases both the training cycle and complexity. Liu et al. [41] leverage historical diagnostic information to improve generation performance by augmenting Large Language Models (LLMs). Nevertheless, they overlook the challenge of aligning medical terminology with detailed visual tokens. Alam et al. [42] combine concept bottleneck models with a multi-agent retrieval-augmented generation system, producing interpretable concept vectors to assist in report generation. Han et al. [43] adopt MedCLIP to extract visual features and retrieve reports. They design a fusion module that integrates image and retrieved report embeddings to model cross-modal information. However, both [42] and [43] fail to address the significant noise present in radiology images, and the ability to capture fine-grained features from blurred scans remains unexplored.

Our approach is distinctly inspired by the real-life medical report writing process of doctors. They keep report generated from traditional decoder in mind and then generate more informational texts by using the key terminologies that they find in the medical images to update the decoder. Although our method demands increased supervision, it is less technically demanding and provides enhanced interactivity, transparency, and explainability.

## III. Methodology

### A. Overview of Framework

**FIGURE 2.**
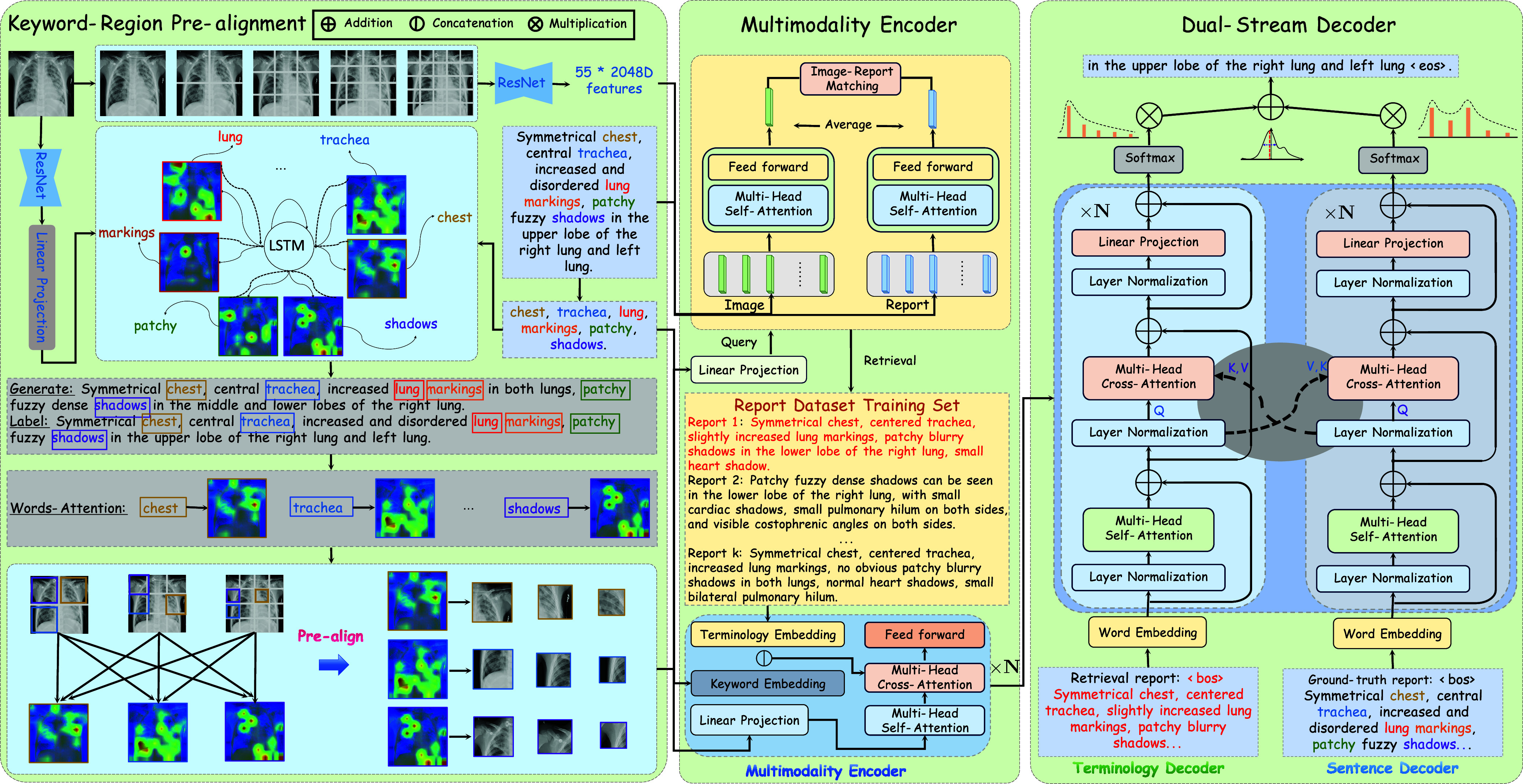
Overview of the proposed Cross-modal Augmented Transformer (CAT): First, Keyword-Region Pre-alignment infers cross-modality mappings between image patches and report tokens, effectively bridging semantic gaps between modalities. Then, a cross-modal retriever is employed to identify additional candidate reports. Subsequently, the encoder is enhanced by multi-modality interaction. Finally, the retrieved reports offer additional semantic cues via cross-branch self-attention.

**FIGURE 3.**
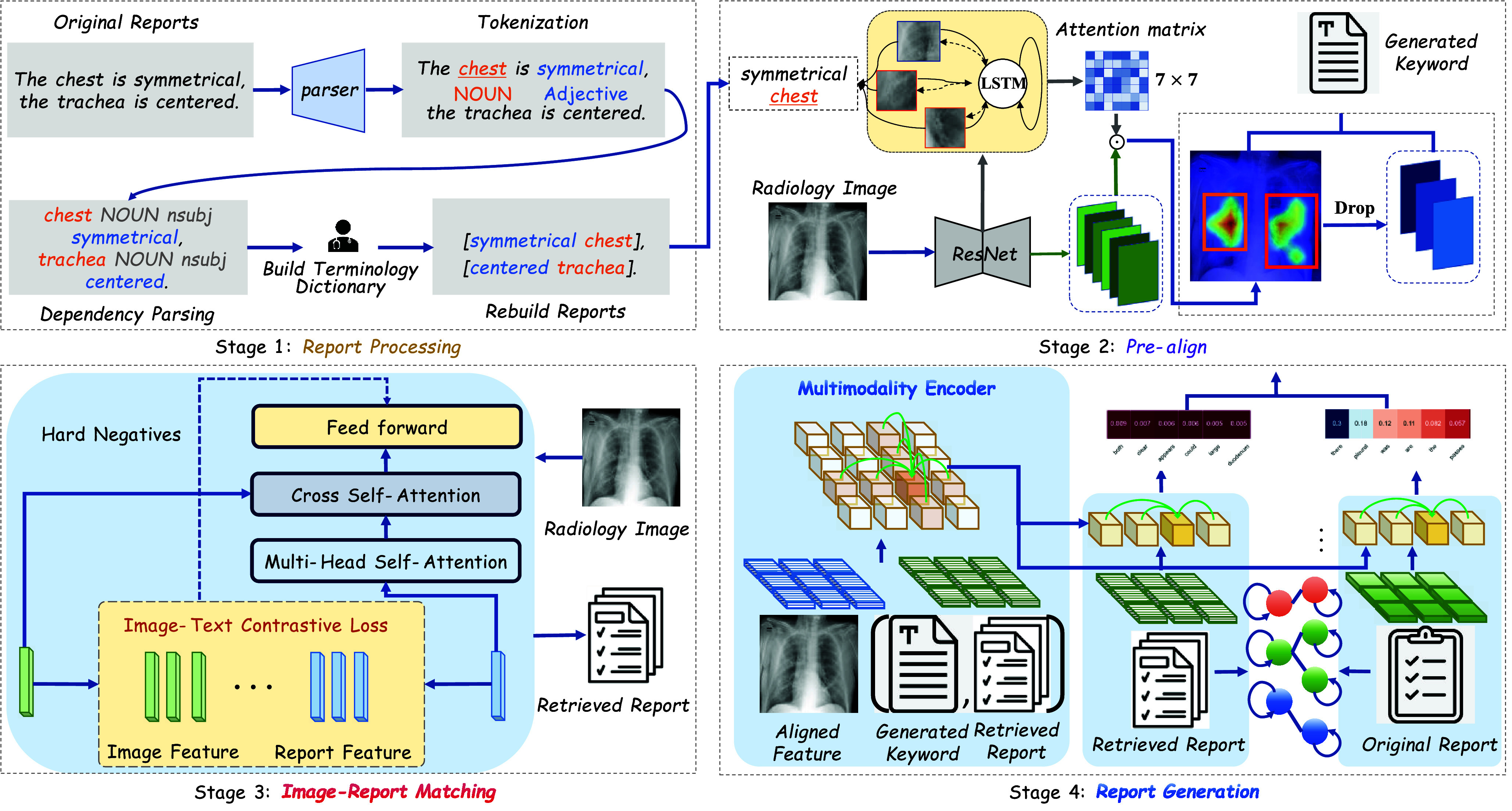
The workflow of the proposed Cross-modal Augmented Transformer.Initially, entities and relations are extracted from the reports. Subsequently, image patches and terminologies are aligned based on attention matrix. Then, a cross-modal retrieval module is trained to find similar reports, and the retrieved reports are utilized as auxiliary information to enhance the encoder. Finally, a dual-stream decoder combines the advantages of retrieved reports and original reports.

The framework of the proposed Cross-modal Augmented Transformer is depicted in Fig. 2. CAT begins with the design of a Keyword-Region Pre-alignment module(KRP) that incorporates the object dropout strategy based on an Attention-based LSTM generation framework. As depicted in Fig. 3, utilizing Key Region Proposal (KRP) enables the capture of content-specific regions. The LSTM-based generation model undergoes pre-training, facilitating the acquisition of a pre-aligned attention matrix. Subsequently, fine-grained features are derived through the element-wise product operation between the initial patch features and the attention matrix. Moreover, the noise within the features is effectively attenuated by prioritizing the scores arranged in ascending order based on the attention matrix. Given the inherent inaccuracies in terminologies and the natural noise present in visual features, a cross-modal encoder is employed to encode the processed features alongside the retrieved terminologies and generated keywords. This operation acknowledges the potential discrepancies between the texts and the actual visual representations, aiming to mitigate the impact of such variances on the model’s performance. Additionally, the structured clinical information inherent in the reports serves to augment the visual representations, thereby enriching the contextual understanding of the images. Finally, a dual-branch Transformer-based decoder is utilized. Cross-modal self-attention interaction occurs not only between visual and textual modalities but also between the two decoding branches, bridging the semantic gap between the retrieval report and the original report. This decoder dynamically evaluates whether the predicted word should be conditioned more heavily on the generated report or the retrieved terminologies. The dual-branch design enhances the generation of meaningful words while simultaneously curbing the excessive generation of template-based words.

### B. Keyword-Region Pre-Alignment Module

Given a medical image, we first cut the image into 55 patches( $1 \times 1, 2 \times 2, 3 \times 3, 4 \times 4, 5 \times 5$). Each patch is encoded using the final layer of ResNet-101, resulting in an output $I_{i}$ with a fixed dimension of 2048 as follows:

\begin{equation*} I=\left \{{{I_{1}, \ldots, I_{i}, \ldots I_{L}}}\right \} \tag {1}\end{equation*}

where $L=55$ represents the total number of vectors. Importantly, each vector is associated with a specific area of the medical image, effectively mapping distinct regions to their corresponding features.

Medical images are predominantly black and white, exhibiting minimal differences among individual images. When feature extraction is performed using convolutional neural networks without prior alignment, the process tends to yield similar features across different images. This similarity in features can introduce biases into the training outcomes. Subsequently, the feature vectors are fed into an attention model for initial training. Through the attention mechanism, we establish connections between each feature and compute their respective attention scores. These scores are then used to generate weight matrices, as denoted below:

\begin{equation*} \alpha _{l}=\left \{{{\alpha _{t 0}, \ldots, \alpha _{t i}, \ldots, \alpha _{t L}}}\right \} \tag {2}\end{equation*}

where $\alpha _{t}$ designates the sought attention matrix and *ti* represents the contribution score of $a_{i}$ at time *t*. The computation process of attention weight is denoted as follows:

\begin{align*} e_{ti}& =f_{att} (a_{i},h_{t-1}) \tag {3}\\ \alpha _{ti}& =\frac {exp(e_{ti})}{\sum _{k=1}^{L} exp(e_{tk})} \tag {4}\end{align*}

Here, $h_{t-1}$ represents the latent vector of the decoder module at time $t-1$, and $f_{att}$ denotes a hidden layer of the LSTM. The attention matrix captures the relationships between regions and medical words, facilitating the extraction of detailed medical information.

Finally, through the dot product of the acquired attention matrix with the visual features, the generation model will further enhance its understanding of medical imaging information by leveraging the fine-grained features. The vital features $I_{f}$ can be derived:

\begin{equation*} I_{f} = \alpha _{t}\cdot I \tag {5}\end{equation*}

One of the significant differences between medical scans and real-world images is the inherent challenge of accurately capturing the target in images. While leveraging comprehensive visual features can mitigate feature loss, it also increases computational complexity. Initially, we introduce a random dropout of certain features with a probability denoted as $p_{obj}$, yet the model sustains commendable performance. However, this method lacks interpretability, merely suggesting computational redundancy in utilizing all features, which could potentially lead to the loss of text-related features. Consequently, we explore a methodology for more refined feature extraction.

Drawing inspiration from the attention matrix, we opt to retain a subset of features for participation in the generation process. This approach allows the model to concentrate more sharply on pertinent features while minimizing the influence of irrelevant elements. Specifically, we introduce an attention object dropout probability $p_{atto}$, indicating the likelihood with which the model discards features. Consequently, we utilize the retained features for training, thereby improving both training efficiency and the accuracy of report generation.

### C. Image-Report Contrastive Learning and Matching

Image-Report Contrastive Learning aims to enhance representations prior to fusion. Drawing inspiration from [44], an association function $l=f_{v}\left ({{\boldsymbol {v}}}\right)^{\top } f_{w}\left ({{\boldsymbol {r}}}\right)$ is defined, where aligned image-text pairs receive stronger association. Here, $f_{v}$ and $f_{r}$ denote linear layers that map the visual and report embeddings to normalized embeddings. The embedding features from two encoders are denoted as $f_{r}^{\prime }\left ({{\boldsymbol {r}^{\prime }}}\right)$ and $f_{v}^{\prime }\left ({{\boldsymbol {v}^{\prime }}}\right)$. In this paper, we define $l(V, R)=f_{v}\left ({{\boldsymbol {v}}}\right)^{\top } f_{r}^{\prime }\left ({{\boldsymbol {r}^{\prime }}}\right)$ and $l(R, V)=f_{r}\left ({{\boldsymbol {r}}}\right)^{\top } f_{v}^{\prime }\left ({{\boldsymbol {v}^{\prime }}}\right)$.

The association score of image-to-report(v2r) and report-to-image(r2v) for each image and report is defined as follow:

\begin{align*} a_{n}^{\mathrm {v} 2 \mathrm {r}}(V) & =\frac {\exp \left ({{l\left ({{V, R_{n}}}\right) / \epsilon }}\right)}{\sum _{n=1}^{N} \exp \left ({{l\left ({{V, R_{n}}}\right) / \epsilon }}\right)} \\ a_{n}^{\mathrm {r} 2 \mathrm {v}}(R) & =\frac {\exp \left ({{l\left ({{R, V_{n}}}\right) / \epsilon }}\right)}{\sum _{n=1}^{N} \exp \left ({{l\left ({{R, V_{n}}}\right) / \epsilon }}\right.} \tag {6}\end{align*}

where $\epsilon $ is a learnable parameter. $s^{\mathrm {v} 2 \mathrm {r}}(V)$ and $s^{\mathrm {r} 2 \mathrm {v}}(R)$ represent the ground-truth association scores. The probabilities of negative pairs and positive pairs are defined as 0 and 1 respectively. The image-report contrastive loss is defined as the cross-entropy C between predicted score $\boldsymbol {g}$ and ground-truth score $\boldsymbol {s}$:

\begin{align*} {\mathcal {L}}_{\mathrm {c}}& =\frac {1}{2} \mathbb {F}_{(V, R) \sim D}\left [{{\!\mathrm {C}\left ({{\boldsymbol {s}^{\mathrm {v} 2 \mathrm {r}}(V), \boldsymbol {g}^{\mathrm {v} 2 \mathrm {r}}(V)}}\right)\!+\!\mathrm {C}\left ({{\!\boldsymbol {s}^{\mathrm {r} 2 \mathrm {v}}(R), \boldsymbol {g}^{\mathrm {r} 2 \mathrm {v}}(R)\!}}\right)\!}}\right ] \tag {7}\end{align*}

As depicted in Stage 3 of Fig. 3, the joint embedding of the image-report pair is derived from the output embedding of cross self-attention. A linear layer combined with softmax is adopted to generate a two-class probability. The matching loss is defined as follows:

\begin{equation*} {\mathcal {L}}_{\mathrm {m}}=\mathbb {F}_{(V, R) \sim D} \mathrm {C}\left ({{\boldsymbol {s}, \boldsymbol {g}(V, R)}}\right) \tag {8}\end{equation*}

### D. Multi-Modality Encoder

In medical datasets, a prevalent visual bias arises due to two primary factors: the limited presence of abnormal regions within the overall image composition and the high degree of similarity among human tissues. To mitigate the influence of this visual bias, $I_{att}$ is condensed into a singular compressed visual token denoted as $I_{c}$. This compressed token is then inputted into the self-attention layer to model the interaction between the visual modality. After that, the retrieved terminology feature $T_{c}$ and generated keyword feature $T_{k}$ are concatenated as $T_{t}$. Then, the interaction between visual and text is operated by a cross self-attention block. This offers dual benefits. Firstly, it ensures that the information from the retrieved report feature $T_{c}$ takes precedence over the input features. Secondly, it adaptively counteracts any inherent noise present within the visual features, particularly in cases where the aligned region features may not be entirely precise.

Following the concatenation process, a position embedding is incorporated into the token embedding. The formulation for this integration can be expressed as follows:

\begin{align*} pos(2 p)& =\sin \left ({{pos / 10000^{2 p /\left ({{d_{\text {model}~} / 2}}\right)}}}\right) \\ pos(2 p+1)& =\cos \left ({{pos / 10000^{2 p /\left ({{d_{\text {model}~} / 2}}\right)}}}\right) \\ pos& =\left [{{pos(2 p); pos(2 p+1)}}\right ] \tag {9}\end{align*}

where *p* symbolizes the dimensions of both the row and column indices, while *pos* denotes their specific position. The subsequent phase entails seamlessly integrating absolute positional data into the conventional scaled dot-product attention mechanism. This integration is designed to augment the model’s capacity to discern between diverse word tokens, thereby enriching its comprehension of the sequential dynamics inherent within the input dataset:

\begin{align*} Q, K, V& =\begin{cases} \displaystyle Q=E_{t} W_{i}^{q}+p o s_{q} \\ \displaystyle K=E_{t} W_{i}^{k}+p o s_{k} \\ \displaystyle V=E_{t} W_{i}^{v} \end{cases} \tag {10}\\ {h}_{t}& =\rm {softmax}\left ({{\frac {Q\left ({{K}}\right)^{T}}{\sqrt {d_{k}}}}}\right) V \tag {11}\end{align*}

The next step involves feeding ${h}_{t}$ into feed-forward networks. This process allows for further transformation and processing of the information encoded in the hidden states:

\begin{equation*} {h}_{t}^{enc}=\text {LN}(\text {FFN}({h}_{t}) + {h}_{t} \tag {12}\end{equation*}

where FFN refers to the feed-forward layer, and LN refers to Layer Normalization. The feed-forward layer consists of two linear layers with ReLU activation, ensuring the transformer’s input and output dimensions remain consistent.

### E. Dual-Stream Decoder

As depicted in the right section of Fig. 2, the language model integrates the self-attention module within both the terminology and report streams to effectively capture the distinct logical structures present in medical reports. Within this framework, the predicted words in a sequence are conditioned on suggestions derived from both branches. Specifically, in the terminology stream, the retrieved terminologies $T_{c}$ are input into the self-attention module to model interactions. Simultaneously, in the report stream, the predicted report $E_{c}$ undergoes processing by the self-attention module to exploit these interactions. Subsequently, cross-modal attention between the encoder and the two streams is employed to establish correlations between the two modalities. For brevity, repeated formulas in this section are omitted. To further bridge the semantic gap between terminologies and reports, cross-branch self-attention interactions are utilized between the two branches. The cross-branch self-attention modules are defined as follows:

\begin{align*} Att_{i}^{tem}(Q_{tem}, K_{rep}, V_{rep})& =\rm {softmax}\left ({{\frac {Q_{tem}, K_{rep}^{T}}{\sqrt {d_{k}}}}}\right) V_{rep} \tag {13}\\ Att_{i}^{rep}(Q_{rep}, K_{tem}, V_{tem})& =\rm {softmax}\left ({{\frac {Q_{rep}, K_{tem}^{T}}{\sqrt {d_{k}}}}}\right) V_{tem} \tag {14}\end{align*}

where *Q*, *K*, and *V* represent the packed d-dimensional query, key, and value vectors, respectively.

Let $\left \{{{y_{1}, y_{2}, \cdots, y_{l}}}\right \}$ denote the predicted reports, where $y_{i}$ represents the index of the *i*-th word in the dictionary. Additionally, let $\left \{{{p_{1}, p_{2}, \cdots, p_{l}}}\right \}$ denote the predicted probabilities of the words in the dictionary, where $p_{i}$ is the probability associated with the *i*-th word. Furthermore, let $p_{i}^{\text {tem}~}$ and $p_{i}^{\text {rep}~}$ represent the output probabilities of the terminology and report streams for the *i*-th word, respectively. The output probabilities from the two branches are computed as follows:

\begin{align*} p_{i}^{tem}& =\rm {softmax}\left ({{\rm {FFN}\left ({{h_{i}^{tem}}}\right) W_{tem}+b_{tem}}}\right) \tag {15}\\ p_{i}^{rep}& =\rm {softmax}\left ({{\rm {FFN}\left ({{h_{i}^{rep}}}\right) W_{rep}+b_{rep}}}\right) \tag {16}\end{align*}

where $h_{i}^{tem}$ and $h_{i}^{rep}$ represent the last hidden state of the each branch.

Then, the probabilities from two branches are combined using the equation $p_{i}=\alpha p_{i}^{\mathrm {tem}}+\beta p_{i}^{\mathrm {rep}}$, where $\alpha $ and $\beta $ are the hyperparameters utilized to balance the outputs of the two-streams. This weighted combination allows us to effectively integrate the information from both the terminology and report streams, ensuring that the final probability reflects the contributions from each source according to their respective importance. In this manner, the final probability $y_{i}$ is computed as:

\begin{equation*} y_{i}=\arg \max _{i} \left ({{\alpha p_{i}^{\text {tem}~}+\beta p_{i}^{\text {rep}~}}}\right) \tag {17}\end{equation*}

where *i* iterates over all possible words in the dictionary. This methodology ensures that the word with the highest cumulative probability is chosen as the predicted output, thereby bolstering the precision and pertinence of the generated reports.

### F. Training

**FIGURE 4.**
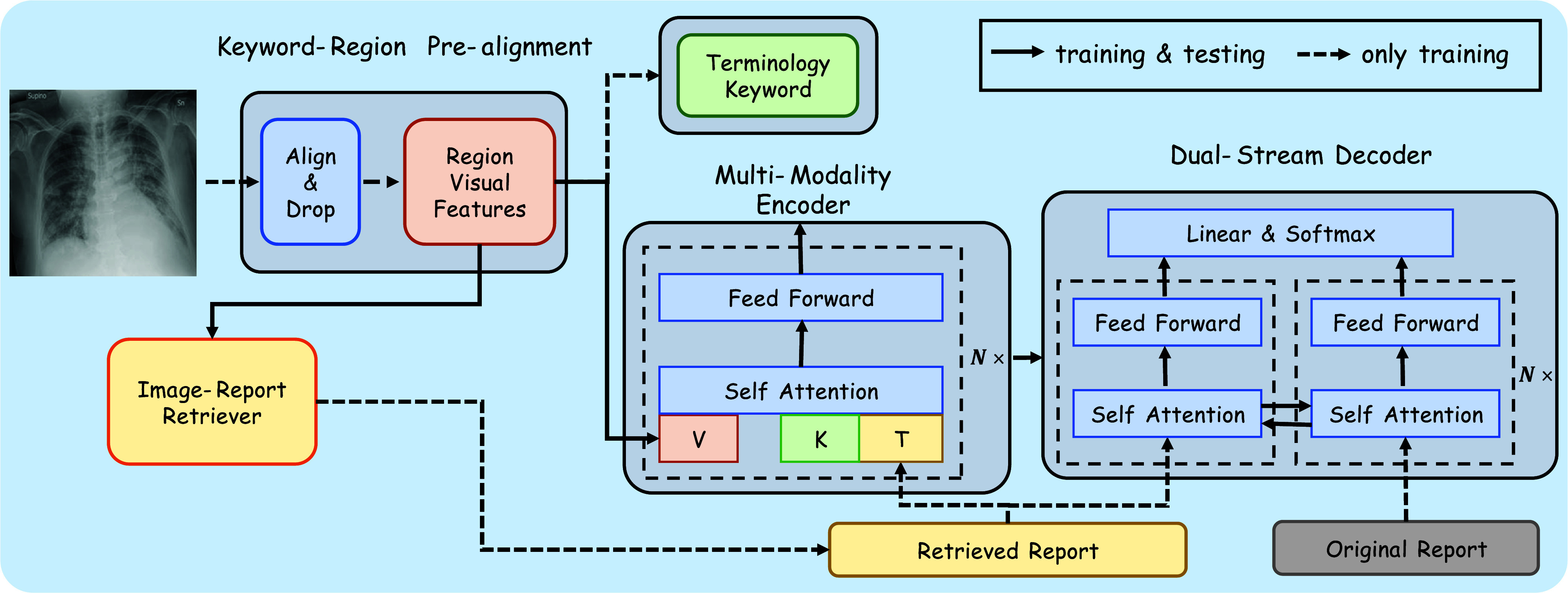
The process of training and testing: the Keyword-RegionPre-alignment Module is first trained to capture visual features corresponding to distinct anatomical regions. Subsequently, a Multi-Modality Encoder enhances the representation of significant abnormal information within the features by combining visual and terminology features. Finally, a Dual-Stream Decoder is employed to produce descriptive sentences for the identified regions, culminating in the generation of the comprehensive report.

As shown in Fig. 4, the model training process comprises two distinct stages. Initially, the training focuses solely on the Keyword-Region Pre-alignment module. Subsequently, the report generation model is trained end-to-end, with all parameters left trainable. During the training of the language model, emphasis is placed on utilizing region visual features that are associated with abnormal terms. This approach operates under the assumption that the region selection module accurately identifies these regions during testing.

Given the ground-truth indices of the preceding $i-1$ words and the concatenation of the two streams, the predictions for the current *i*-th word can be obtained. Given the ground truth report $r=\left \{{{y_{1}^{*}, y_{2}^{*}, \ldots, y_{s}^{*}}}\right \}$, we train the generation model by minimizing the cross-entropy loss:

\begin{equation*} {\mathcal {L}}_{\mathrm {CE}}(\theta)=-\sum _{i=1}^{s} \log \left ({{p_{\theta }\left ({{y_{i}^{*} \mid y_{1: i-1}^{*}}}\right)}}\right) \tag {18}\end{equation*}

## IV. Experiments

### A. Experiment Details

#### 1) Datasets

In this paper, we leverage two public medical report generation datasets: MIMIC-CXR [1], the largest repository of its kind with a staggering 377,110 chest X-ray images and 227,835 detailed clinical narratives; and ROCO [47], which offers a diverse collection of over 81,000 images spanning radiological and non-radiological domains. The MIMIC-CXR dataset provides comprehensive diagnostic insights for each case, ensuring a robust evaluation based on official data splits. Meanwhile, ROCO boasts a vast array of non-radiological images, significantly amplifying the complexity in model design. All tokens are converted to lowercase, and those with a frequency of occurrence less than five are removed. Additionally, we incorporate the [PAD], [SOS], [EOS], and [UNK] tags, with indices assigned as 0, 1, 2, and 3, respectively, into the vocabulary.

#### 2) Evaluation Metrics

In this paper, we adhere to the official metrics for evaluation, including BLEU [48], METEOR [49], ROUGE-L [50], and CIDEr [51], to assess the quality of the predicted reports at the word level.

### B. Experiment Results

To validate the effectiveness of the proposed method, we compare it with eight competitive methods, as reported in Table 1. The upper part of the table presents the results of classical image captioning methods, while the lower part displays the medical report generation methods. For these methods, we utilize a publicly released codebase [46] on the IU X-Ray dataset and re-run them on the ROCO dataset under the same experimental settings. As observed, our CAT model achieves superior results across all metrics on both datasets. Specifically, the CAT model improves the absolute CIDEr score by 10.6% and 1.4% compared to the strong competitive method PPKED [34] on the two datasets, respectively. Furthermore, the proposed CAT demonstrates a significant enhancement over the state-of-the-art model, METransformer [46], with the CIDEr score increasing from 43.5% to 45.7% and from 10.5% to 11.8% on the two datasets, respectively. This improvement can be attributed to the use of aligned visual features, which reduce noise and lower the structural requirements of the network compared to complex self-attention mechanisms. In comparison to competitive image captioning models such as RSTNet [6], our model continues to exhibit notable superiority, with the CIDEr score rising from 35.6% to 45.7% and from 9.5% to 11.8% across the two datasets, respectively. This advantage is primarily due to the fact that decoders in general domains have not adequately considered the unique logical structure inherent in medical reports, leading to significant performance degradation in one-stop generation modes. Overall, the proposed CAT model achieves state-of-the-art results, outperforming all other models on both datasets.

**TABLE 1:** Performance Comparison (%) With State-of-the-Art Methods on IU X-Ray and ROCO Datasets. BLEU-1, BLEU-2, BLEU-3, BLEU-4, METEOR, ROUGE-L and CIDEr are Reported

Dataset	Methods	Ref	BLEU-1	BLEU-2	BLEU-3	BLEU-4	METEOR	ROUGE-L	CIDEr
IU X-Ray	Updown [23]	CVPR18	37.5	23.7	16.7	12.3	-	31.9	33.6
	AoA [5]	CVPR19	38.2	24.5	16.6	12.7	16.8	32.1	33.5
	Mesh-Memory [8]	CVPR20	40.2	28.4	16.8	14.3	17.0	32.8	33.2
	RSTNet [6]	CVPR21	41.0	28.9	18.3	15.7	17.7	35.2	35.6
	R2gen [13]	EMNLP20	47.0	30.4	21.9	16.5	18.7	37.1	-
	PPKED [34]	CVPR21	48.3	31.5	22.4	16.8	19.0	37.6	35.1
	R2genCMN [45]	arxiv22	47.5	30.9	22.2	17.0	19.1	37.5	-
	METransformer [46]	CVPR23	48.3	32.2	22.8	17.2	19.2	38.0	43.5
	CAT (ours)	-	49.1	32.7	23.3	17.6	19.5	38.3	45.7
ROCO	Updown [23]	CVPR18	18.2	9.7	4.4	2.1	6.3	15.2	8.9
	AoA [5]	CVPR19	17.9	9.3	4.3	2.0	6.2	15.2	9.0
	Mesh-Memory [8]	CVPR20	18.2	9.3	4.2	2.1	6.0	15.3	9.2
	RSTNet [6]	CVPR21	18.3	9.9	4.6	2.2	6.4	15.5	9.5
	R2gen [13]	EMNLP20	18.6	10.2	4.8	2.9	6.8	16.1	9.8
	PPKED [34]	CVPR21	19.1	10.5	5.0	3.1	7.2	16.5	10.4
	R2genCMN [45]	arxiv22	19.3	10.4	4.9	3.0	7.1	16.4	10.2
	METransformer [46]	CVPR23	19.5	10.7	5.1	3.2	7.3	16.6	10.5
	CAT (ours)	-	20.4	11.0	5.5	3.6	7.7	17.2	11.8

### C. Ablative Analysis

To thoroughly evaluate the impact of the proposed Keyword-Region Pre-alignment (KRP) module and the Dual-Stream Decoder (DSD), we conduct an ablation study comparing various variants of the CAT model, as shown in Table 2. We begin with a baseline model that utilizes a standard Multi-Head Self-Attention (MHSA) architecture without any modifications. Subsequently, we integrate the KRP module and the DSD module into the baseline model, respectively. Finally, we combine both the KRP and DSD modules into the baseline model to construct our CAT model. In the experiments, the integration of the KRP and DSD modules yields significant improvements over the baseline transformer model, with the CIDEr score increasing from 33.0% to 41.2% and from 33.0% to 41.6% on the IU X-Ray dataset, and from 8.9% to 10.4% and from 8.9% to 10.6% on the ROCO dataset, respectively. We hypothesize that these performance improvements may be attributed to the alignment emphasizing keywords during report generation, as the alignment features correspond with medical terminologies. While the KRP module shows incremental improvements across various metrics, its synergistic benefits are more pronounced when integrated with the DSD. The diversified decoding branches of the DSD align better with the complex compositional structure of medical reports. Moreover, the combined use of both modules demonstrates a substantial enhancement, underscoring their complementary nature.

**TABLE 2:** Ablation Experiments (%) on IU X-Ray and ROCO Datasets

$No$.	Methods	Modules			IU X-Ray		ROCO	
		KRP	ME	DSD	BLEU-4	CIDEr	BLEU-4	CIDEr
1	MHSA				14.0 ( $3.6\downarrow $)	33.0 ( $12.7\downarrow $)	2.0 ( $1.6\downarrow $)	8.9 ( $2.9\downarrow $)
2	MHSA $with$ KRP	✓			16.9 ( $0.7\downarrow $)	41.2 ( $4.5\downarrow $)	3.2 ( $0.4\downarrow $)	10.4 ( $1.4\downarrow $)
2	MHSA $with$ ME		✓		15.7 ( $1.9\downarrow $)	39.4 ( $6.3\downarrow $)	2.9 ( $0.7\downarrow $)	10.0 ( $1.8\downarrow $)
3	MHSA $with$ DSD			✓	16.5 ( $1.1\downarrow $)	41.6 ( $4.1\downarrow $)	3.3 ( $0.3\downarrow $)	10.6 ( $1.2\downarrow $)
4	CAT	✓	✓	✓	17.6	45.7	3.6	11.8

#### 1) Effect of Keyword-Region Pre-Alignment

**FIGURE 5.**
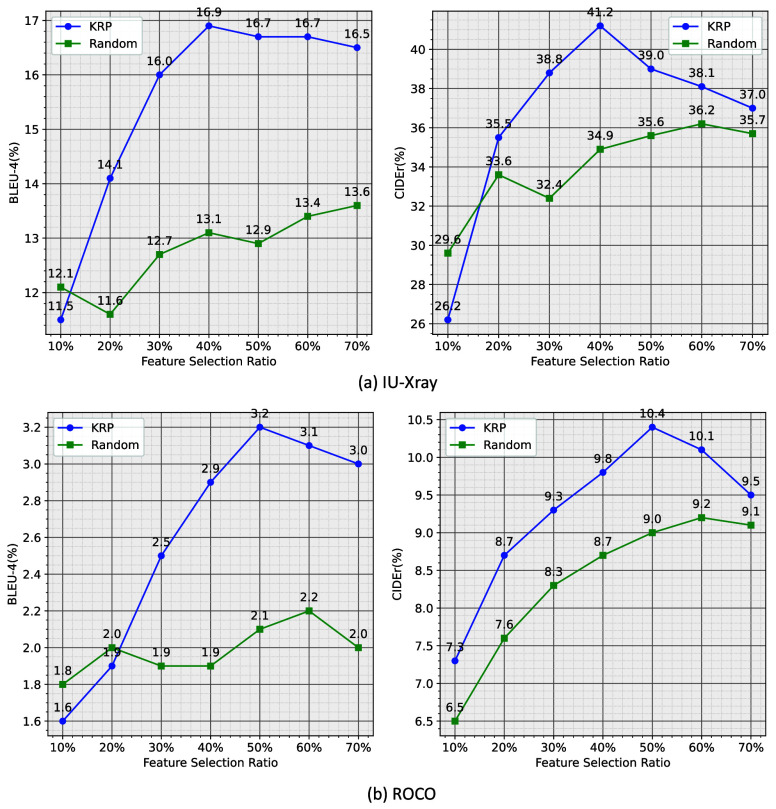
Effect of feature selection ratio on generation performance (%). KRP (blue line) and Random drop (green line) are reported, with corresponding high values annotated on the figure.

To thoroughly evaluate the effectiveness of pre-alignment, we compare its performance with random dropout. Random dropout involves randomly omitting certain areas without assessing each grid feature’s contribution to report generation. Fig. 5 illustrates that the Keyword-Region Pre-alignment module consistently outperforms random dropout across all metrics and datasets. This improvement is attributed to the fact that random dropout may inadvertently discard visually relevant information in the text. In contrast, the Keyword-Region Pre-alignment module is designed to more accurately filter out visually irrelevant content. Higher feature selection ratios generally correlate with performance improvements. Notably, once the feature selection ratio exceeds 40%, performance metrics exhibit minimal variation, simplifying the practical selection of the dropout rate in KRP. Overall, the results suggest that increasing the feature selection ratio initially enhances report generation performance up to a threshold value, after which further increases lead to a sudden decline. This decline indicates the presence of noise in medical images that interferes with the Keyword-Region Pre-alignment module’s performance.

#### 2) Effect of Dual-Stream Decoder

In comparison to the traditional single-branch decoder, our dual-stream decoder significantly enhances the base model’s performance. Specifically, employing the dual-stream decoder yields an 8.6% and 1.7% increase in the CIDEr metric across two datasets. This highlights the effectiveness and necessity of implementing an alternating decoding mode for report generation. Medical reports are inherently structured and do not follow a conventional generation process. Instead, they are contextualized with essential medical terminology and require a logical structure. Therefore, contextual logic must be meticulously integrated throughout the report generation process.

**FIGURE 6.**
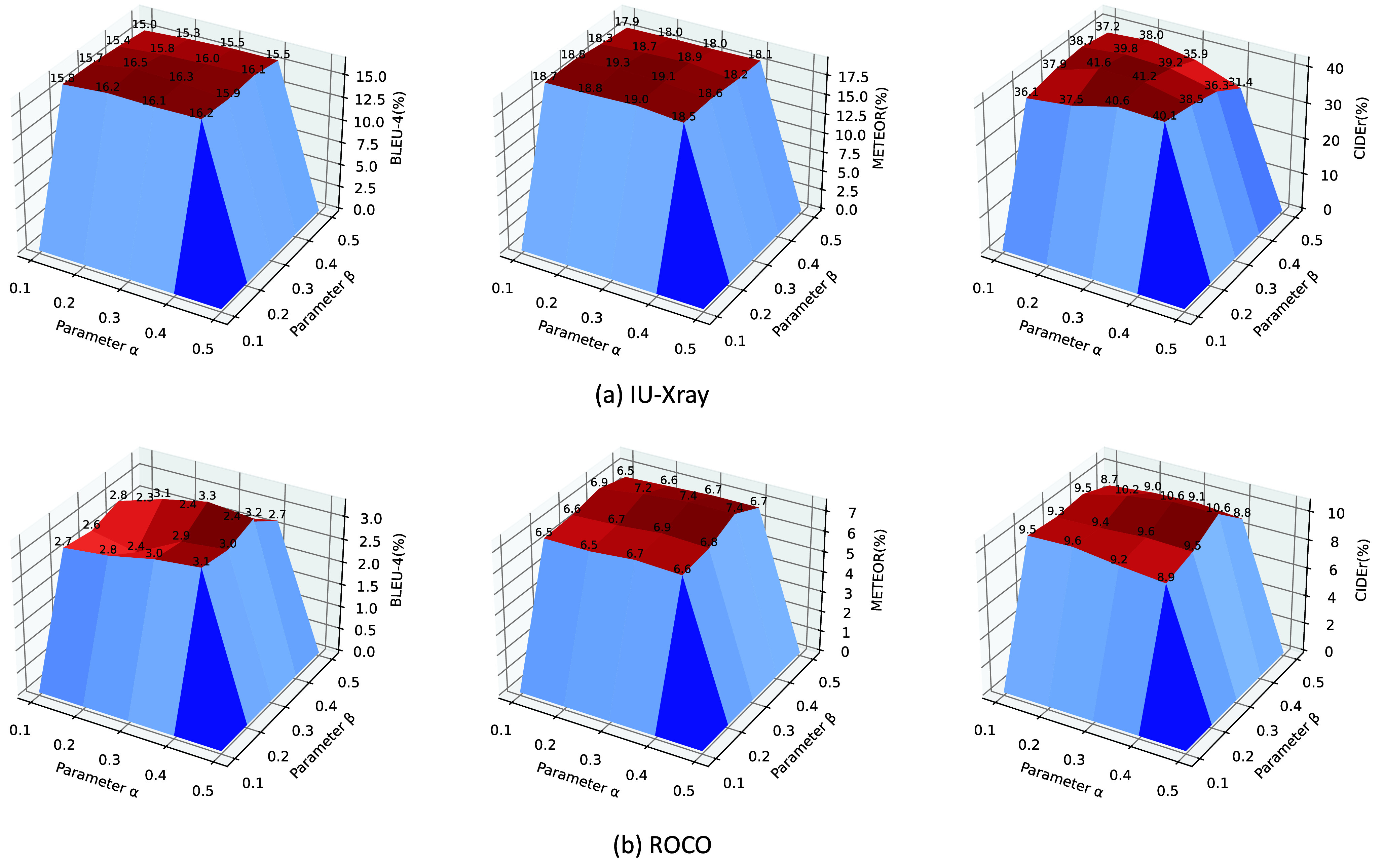
Effect of control coefficients $\alpha $ and $\beta $. (a) and (b) record how BLEU-4, METEOR and CIDEr change with $\alpha $ and $\beta $ on both datasets (%). Brighter colors indicate higher metrics, with corresponding high values annotated on the figure (best viewed in colors).

To investigate the impact of varying weights on each branch, we conducted ablation studies evaluating the proposed dual-stream decoder with parameter selections of 0.1, 0.2, 0.3, 0.4, and 0.5. The experimental findings depicted in Fig. 6 underscore the importance of leveraging both terminology and decoding branches’ strengths. Solely relying on either the standard decoding branch or the terminology branch results in excessive generation of medical terms and template sentences.

**FIGURE 7.**
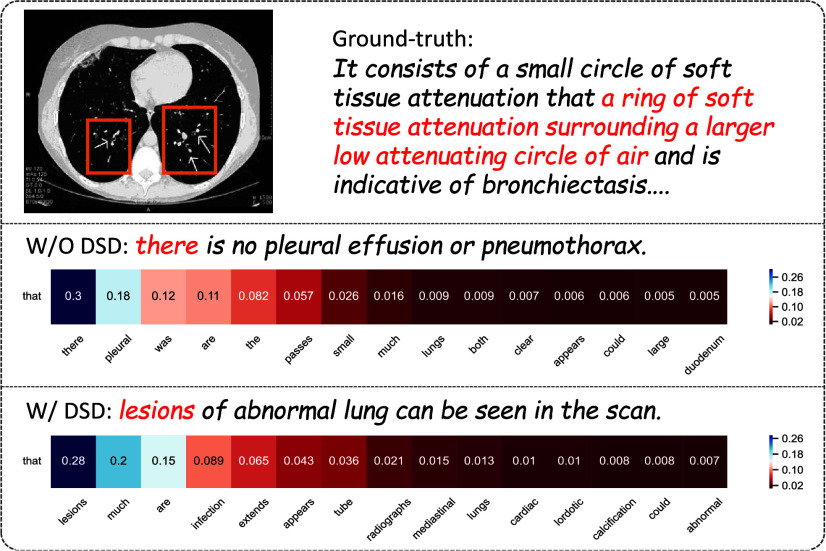
Comparison of the top-15 word candidates generated by the model without Dual-Stream Decoder (W/O DSD) and the model with Dual-Stream Decoder (W/DSD) reveals significant improvements. The conventional decoder often produced irrelevant words like “there”, “was”, and “are” due to template bias. Integration of the Dual-Stream Decoder mitigates this issue, enhancing the generation of specialized terminologies such as “lesions” and “infection”.

We also visually demonstrate the word generation process before and after employing the dual-stream decoder. As illustrated in Fig. 7, prior to utilizing the dual-stream decoder, the model attends to not only medical terms but also other template words, which lacks meaningful context. After using the dual-stream decoder, there is a heightened focus on terminology prediction, leading to more precise generation.

### D. Qualitative Analysis

**FIGURE 8.**
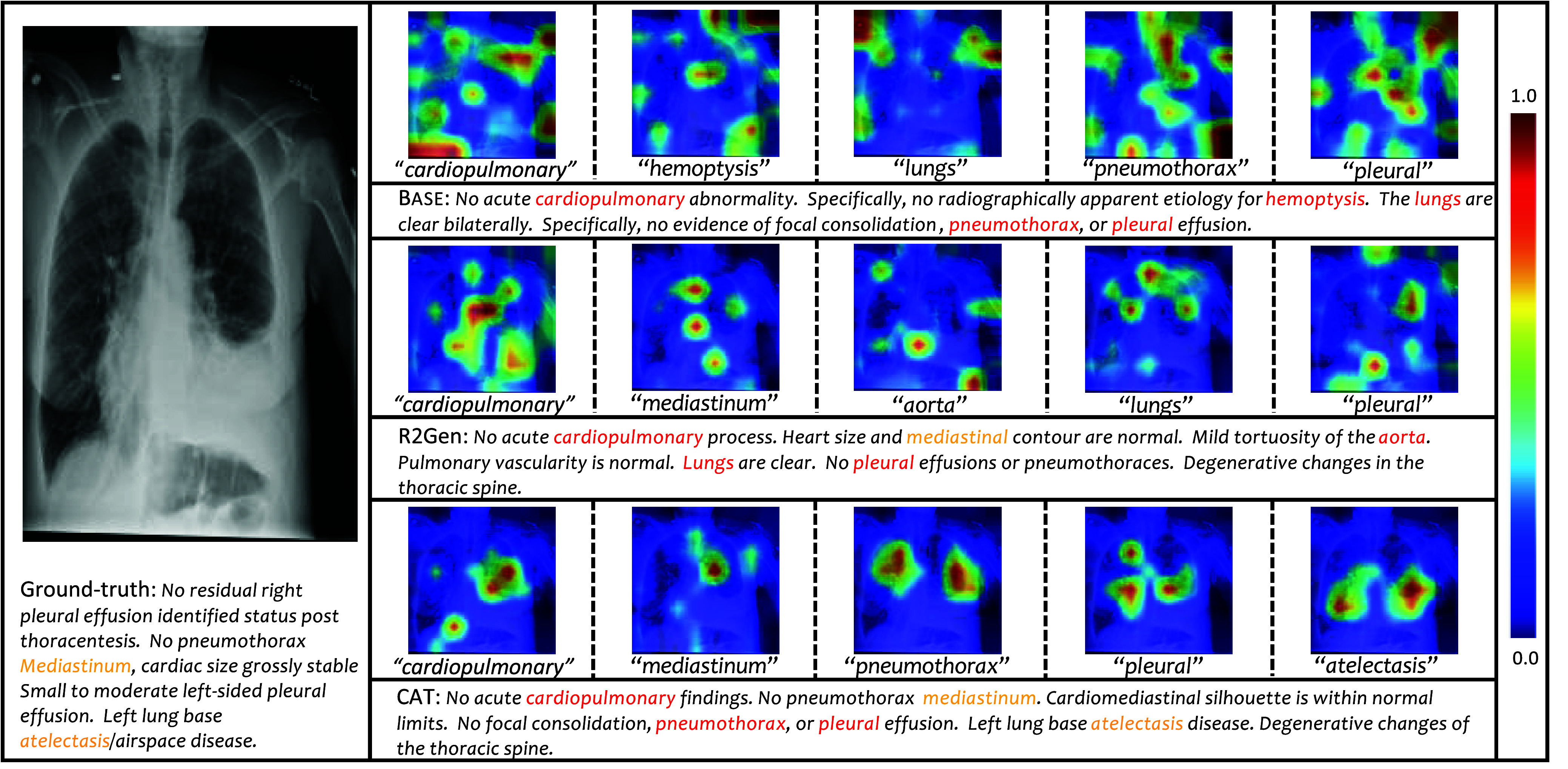
Visualization of attention weights for each image region concerning the generated words on the IU-Xray dataset. In the visual heatmaps, warmer colors denote higher model attention, whereas cooler colors indicate lower attention levels. (best viewed in colors).

**FIGURE 9.**
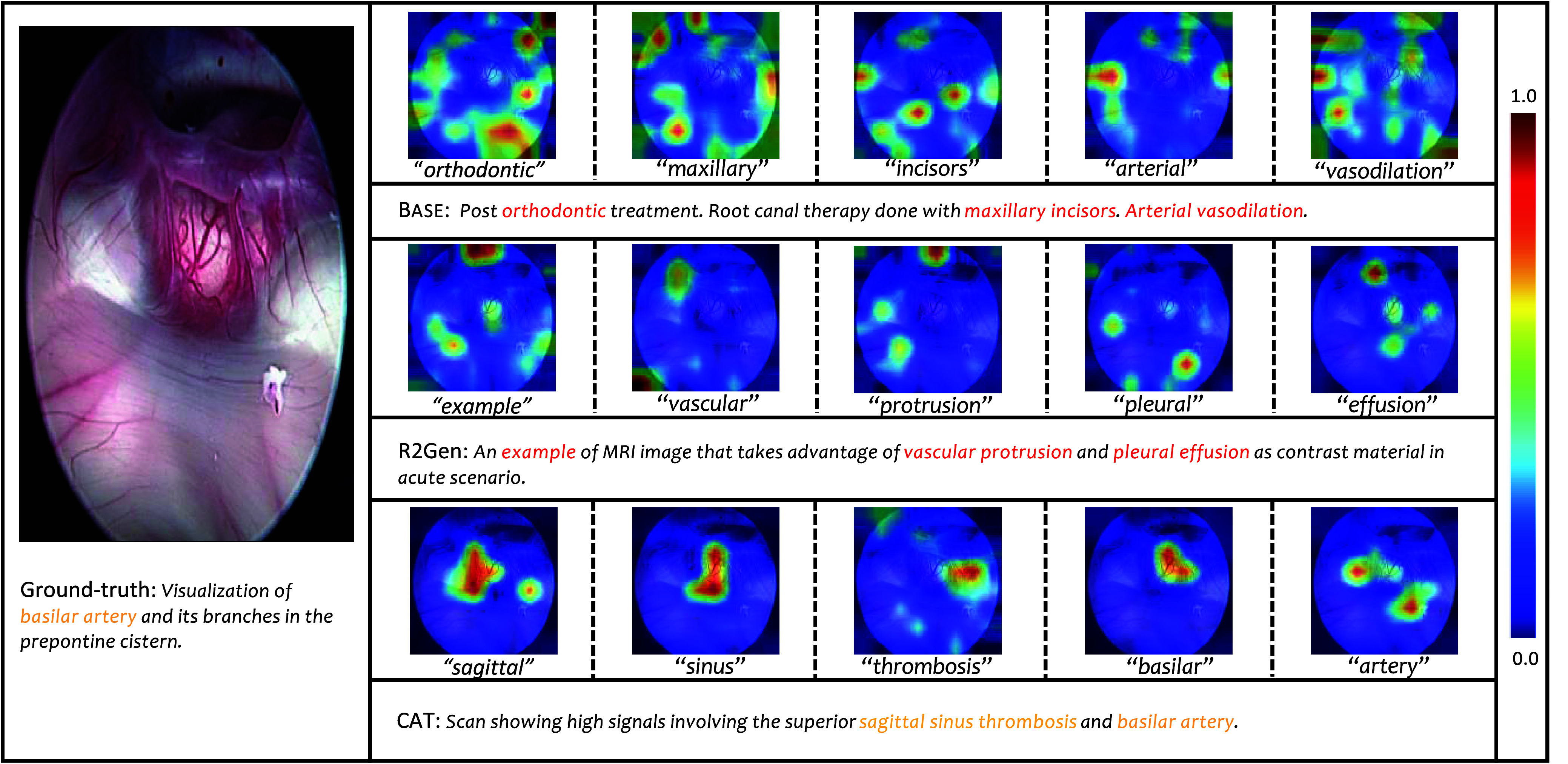
Visualization of attention weights for each image region concerning the generated words on the ROCO dataset.

To gain insights, we visualize the image regions predominantly attended by each terminology word in Fig. 8 and Fig. 9 via exploration of the attention between the learned terminology word embeddings and the visual tokens. For clarity, only the most attended image regions are depicted. It is observed that each terminology word focuses on distinct and crucial image regions. In comparison to the conventional Transformer model (BASE) and the competitive R2Gen model, the proposed CAT model exhibits enhanced focus on the target organ with lesions. In Fig. 8, the image region attended by the word “atelectasis” corresponds to lung collapse, resulting in decreased gas content, offering valuable clinical information. In Fig. 9, the conventional Transformer model (BASE) is affected by feature noise and fails to effectively identify lesion areas, whereas the proposed model can perform meaningful segmentation on tissues such as “artery”. This improvement is primarily attributed to the pre-alignment operation, which strengthens the association between abnormal terms and visual content.

**FIGURE 10.**
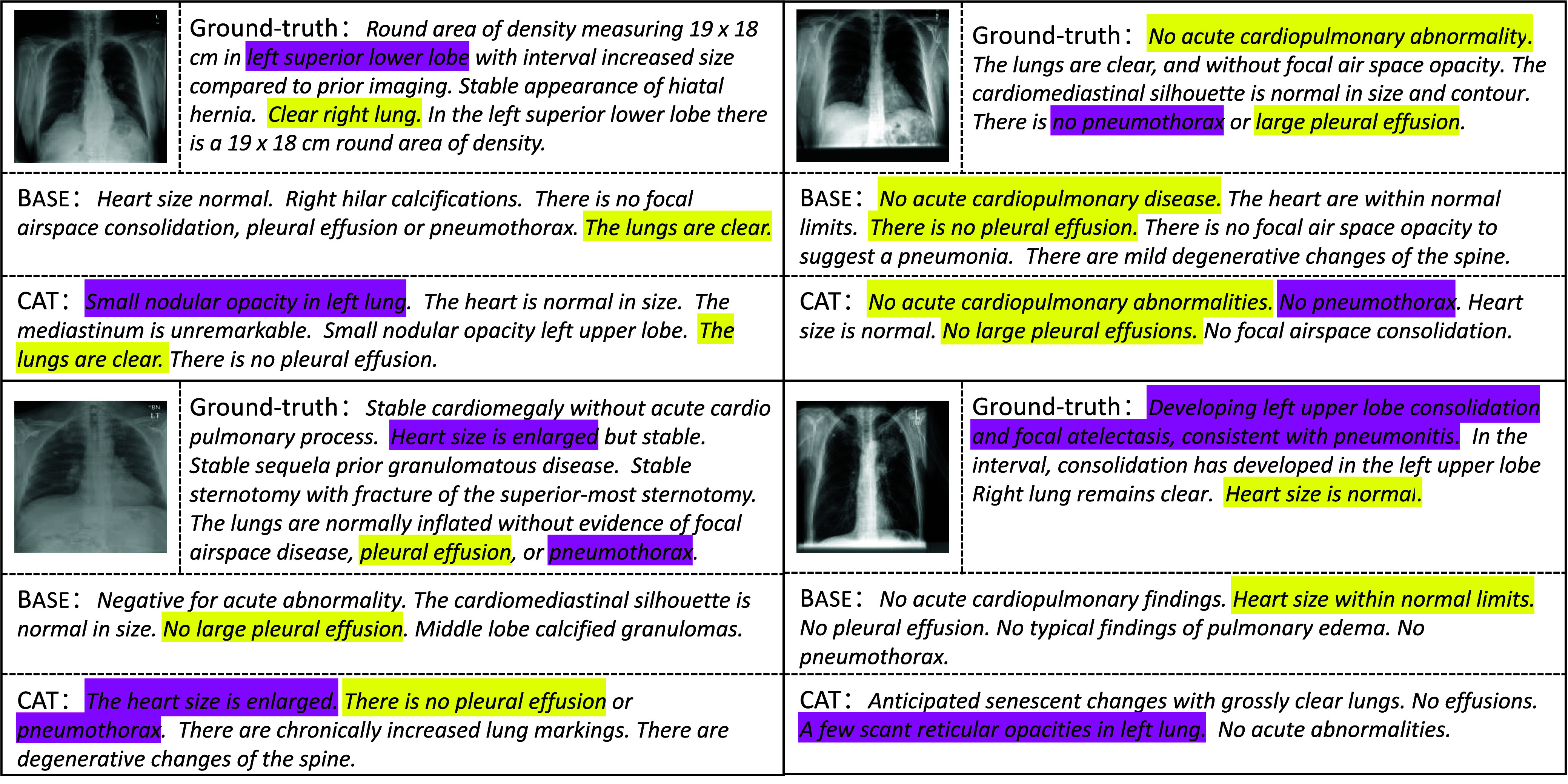
Qualitative analysis of the generated examples on IU-Xray dataset.Purple indicates correct matches produced solely by CAT, while yellow denotes correct matches produced by both the base model and CAT. (best viewed in colors).

Moreover, the proposed model effectively reduces the generation of meaningless templates. For instance, in Fig. 10, the conventional Transformer model tends to produce templated but meaningless sentences like “There is no focal airspace consolidation, pleural effusion, or pneumothorax.” In contrast, the proposed model generates more clinically valuable abnormal descriptions such as “Small nodular opacity in the left lung.” This improvement stems from the dual-branch structure’s ability to adaptively prioritize the generation of medical terms over generic templates, unlike traditional single decoders.

## V. Discussion on Practical Application

### A. Experiments on Clinical Data

We validate the robustness and generation efficiency of the proposed model using a dataset of pneumonia patient records collected from our partner hospitals. It is important to note that due to ethical procedures, we are currently limited to obtaining data from only 2,000 patients. To ensure the originality of the data, we refrain from any cleaning processes in the original reports, intentionally retaining certain noisy elements, such as recommendations for further CT or MRI examinations. This approach allows us to assess the model’s generation performance in environments characterized by noise. The data is divided into training and testing sets at an 8:2 ratio, ensuring that only words occurring more than five times are retained. To accommodate the limitations of GPU memory, we set the batch size to a maximum of 10. The model is trained using two NVIDIA RTX TITAN GPUs to optimize processing efficiency and performance. It is important to emphasize that we believe the efficiency of the model in real-world applications should be assessed based on testing speed rather than training speed. In practical scenarios, we typically fine-tune the model based on a pre-trained version rather than training from scratch. The proposed CAT model demonstrates comparable report quality to competitive report generation models on real-world clinical data, while also achieving a notable speed improvement. The testing time for each batch of the AOA [5] model is 303 ms. Using this benchmark, we assess the testing speed of several models, including Mesh Memory [8], R2Gen [13], R2GenCMN [45], and METransformer [46] on the server. The results reveal that the efficiency of the proposed model exceeds that of most other models, primarily due to the alignment operation, which reduces the influence of noise features on report generation. However, the efficiency of our model is not as high as that of METransformer. This difference can be attributed to the more complex decoder design of our model, which requires two branches to dynamically weigh the fusion results.

**TABLE 3:** Performance Comparisons (%) Based on the Collected Clinical Data are Presented. The Abbreviations B-1, B-2, B-3, B-4, M, R, and C Denote the BLEU [48], METEOR [49], ROUGE-L [50], and CIDEr-D [51] Metrics, Respectively

Dataset	Models	Ref	B-1	B-2	B-3	B-4	M	R	C	Latency	Speedup
Clincial Data	AoA [5]	CVPR19	51.5	47.6	42.5	38.2	30.5	51.7	17.3	301 ms	1.00 $\times$
	Mesh-Memory [8]	CVPR20	53.3	48.2	44.3	39.6	31.0	52.3	17.9	251 ms	1.19 $\times$
	R2Gen [13]	EMNLP20	53.1	47.9	44.5	38.9	30.7	52.0	-	276 ms	1.09 $\times$
	R2GenCMN [45]	arxiv22	53.9	48.6	44.7	39.5	31.2	52.5	-	282 ms	1.06 $\times$
	METransformer [46]	CVPR23	55.1	49.2	45.0	39.9	31.5	52.8	19.2	263 ms	1.14 $\times$
	CAT (ours)	-	56.3	49.7	45.4	40.2	31.8	52.8	20.3	270 ms	1.11 $\times$

### B. Impacts on Rare Samples

**FIGURE 11.**
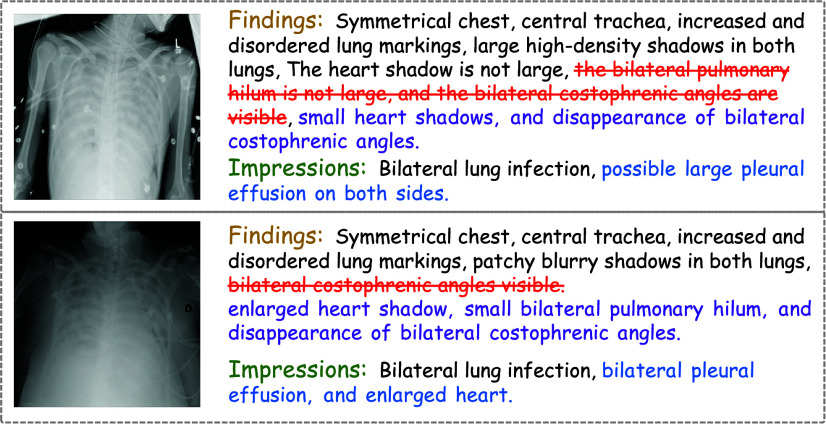
Analysis of the rare generated samples for “bilateral pleural effusion”.

Compared to pulmonary infections and inflammation, the sample size of pleural effusion is much smaller. To verify the generation effect on the rare samples, we choose to generate reports for X-rays with pleural effusion. The results of this generation are presented in Fig. 11. The results indicate that the model’s generation effect on rare samples, such as “bilateral pleural effusion,” is not optimal. This limitation primarily arises because the model struggles to effectively train on abnormal terms found in rare cases. Consequently, while alignment module can generate keywords associated with more common conditions like “lung infections” and “lung inflammation”, it fails to adequately learn and recognize anomalies such as “pleural effusion”.

### C. Limitations

**FIGURE 12.**
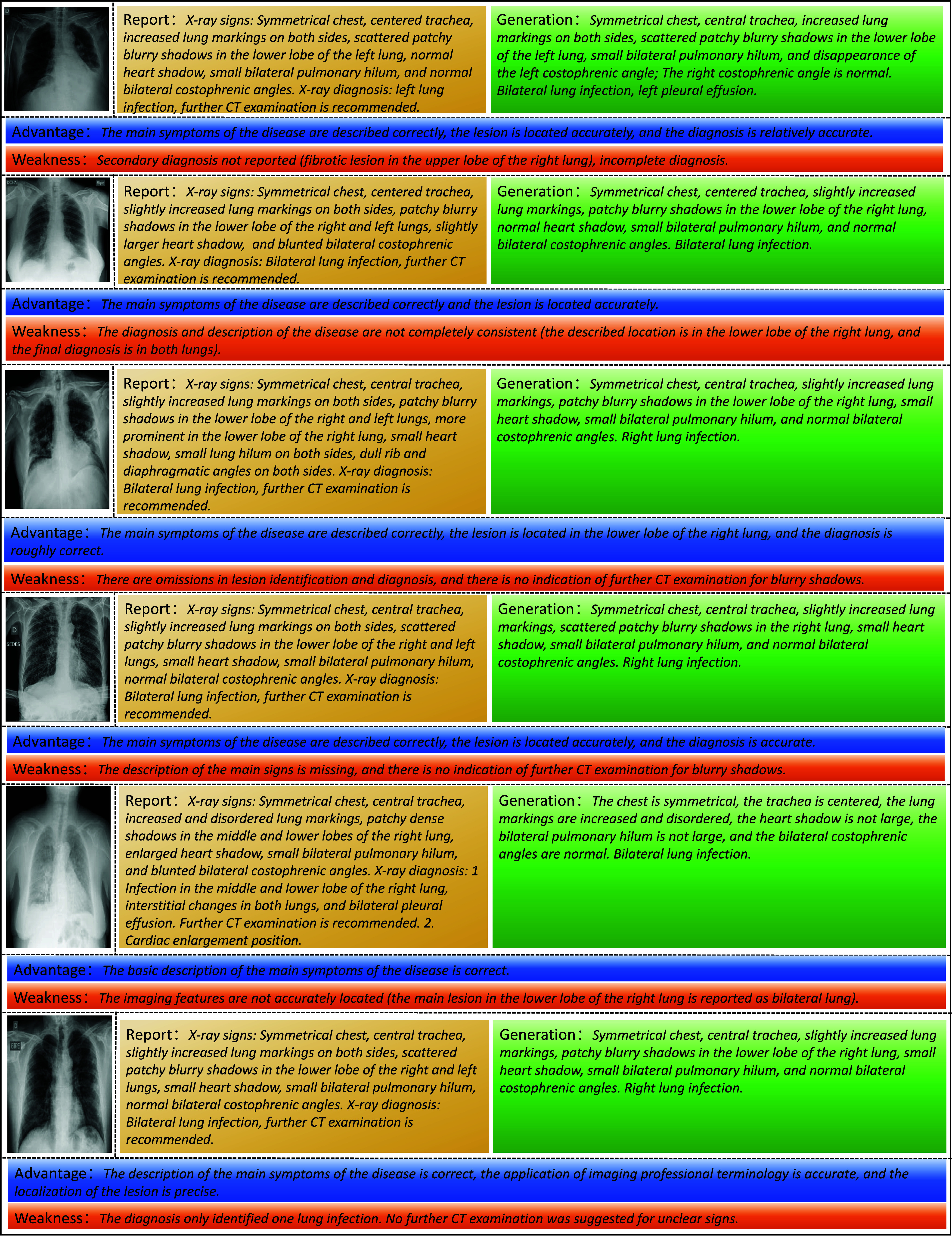
Feedback regarding the generated reports received from radiologists.

As a data-driven framework, the proposed CAT model exhibits sensitivity to the training data distribution it has encountered. As a result, it may struggle when faced with more intricate medical scenarios. This challenge primarily arises from the disparities in data distributions between controlled laboratory environments and real-world contexts. We have also invited a panel of five experienced radiologists to evaluate the generated reports from multiple perspectives. Due to space constraints, we present six selected examples in Fig. 12. Observing these cases, it is evident that our proposed model is capable of describing common, significant symptoms; however, it struggles to accurately capture more subtle symptoms. From an ethical standpoint, the proposed model is currently limited to serving as a reference tool for diagnosis and cannot replace human doctors. This also proves that there is still a considerable gap between the current framework and its implementation in real-world applications. Furthermore, the non-end-to-end design complicates the direct integration of models into tools or extensive language frameworks.

### D. Broader Impacts

In future work, we intend to broaden our efforts to collect real pneumonia X-ray data from our partner hospitals. This will involve acquiring higher-resolution X-ray scans, standardizing disease classifications, verifying the authenticity of reports, and annotating fine-grained bounding boxes to facilitate further detailed vision-language pre-training. Additionally, we aim to develop a more lightweight end-to-end model that effectively combines alignment and report generation, which represents a key research goal moving forward. We also foresee a trend in capturing medical images using mobile phones to provide instant diagnostic reports. However, the limitations of mobile device performance pose challenges for deploying large-scale pre-trained models. Consequently, creating a lightweight pre-trained model is essential for advancing medical mobility and addressing the needs and constraints associated with mobile platforms.

## VI. Conclusion

In this paper, we introduce an effective cross-modal transformer for medical report generation. To capture relevant content features beforehand, we propose an information extraction scheme to align crucial medical terms with significant regions. The aligned terminologies from this module are then injected into visual features for report generation. Finally, we design an alternative dual-stream decoder to dynamically determine whether the target word should be conditioned on the template sentence or the terminology. Experimental results indicate that the proposed model outperforms existing approaches on both laboratory datasets and collected clinical data.
